# Reduction and ring fixation of instable C1 fractures with monoaxial pedicle screws

**DOI:** 10.1007/s00402-017-2737-4

**Published:** 2017-06-26

**Authors:** Rainer Gumpert, Thomas Poglitsch, Renate Krassnig, Rudolf Pranzl, Paul Puchwein

**Affiliations:** 1 “Mozartpraxis”, Raum für Gesundheit, Mozartgasse 4, 8010 Graz, Austria; 2 Department of Orthopedic and Traumatology, Medical University Graz (MUG), Auenbruggerplatz 5, 8036 Graz, Austria; 3 Unfallkrankenhaus Klagenfurt, Waidmannsdorfer Straße 35, 9020 Klagenfurt, Austria

**Keywords:** C1 Jefferson fracture, Unstable C1 fracture, Ring fixation, Monoaxial pedicle screws, Lateral mass screws

## Abstract

**Introduction:**

Ring fixation of C1 can be performed using pedicle screws and a rod in case of unstable Jefferson or lateral mass fractures of C1.

**Materials and methods:**

In a case series of three patients, we stabilized C1 fractures surgically using a modified technique of C1 ring fixation by using monoaxial instead of polyaxial screws. Functional outcome and pain was recorded postoperatively.

**Results:**

In this very small case series, we observed good results concerning pain and functional outcome. All fractures were bony healed within 13 weeks. In one case, a screw penetrated the spinal canal and had to be repositioned. A mild irritation of C2 nerve root occurred in two cases postoperatively.

**Conclusion:**

C1 Ring fusion with monoaxial screws provides a good ability to reduce the fracture indirectly by the screws and the rod itself.

## Introduction

Jefferson fractures are burst fractures of the first cervical vertebra and uncommon [1–3]. Whereas the classical Jefferson fracture involves both arches and both sides, uni- or bilateral lateral mass fractures can either be stable or unstable [1]. The presence of an intact transverse ligament is determining for the stability of the fracture [1, 4, 5]. Whereas stable fracture can be treated conservatively, unstable fractures are usually treated surgically [4]. CT scans are commonly used for proper diagnosis. Separation of lateral masses or dislocations of more than 7 mm have to be considered as unstable and should therefore be treated surgically [4, 5]. Beside techniques resulting in a fusion of the first two cervical vertebrae, procedures maintaining the motion in this segment are becoming more popular [6–8]. Some few publications can be found about lateral mass ring fixation—usually performed with polyaxial pedicle screws and a rod [6, 7]. Using monoaxial pedicle screws, we have modified this technique in order to get a better reduction and probably a greater stability.

## Materials and methods

Between 2010 and 2012, three patients with an unstable Jefferson fracture were treated operatively at our institution using this new technique. In all patients, a posterior approach and ring osteosynthesis with monoaxial pedicle screws and a rod was performed. Follow-up examinations were planned at day 14, after 6 weeks and after 3 months. Implants were removed in two cases. Final follow-up was performed at least 1 year after hardware removal. One patient was not contactable for the final follow-up.

### Surgical technique

**Fig. 1:**
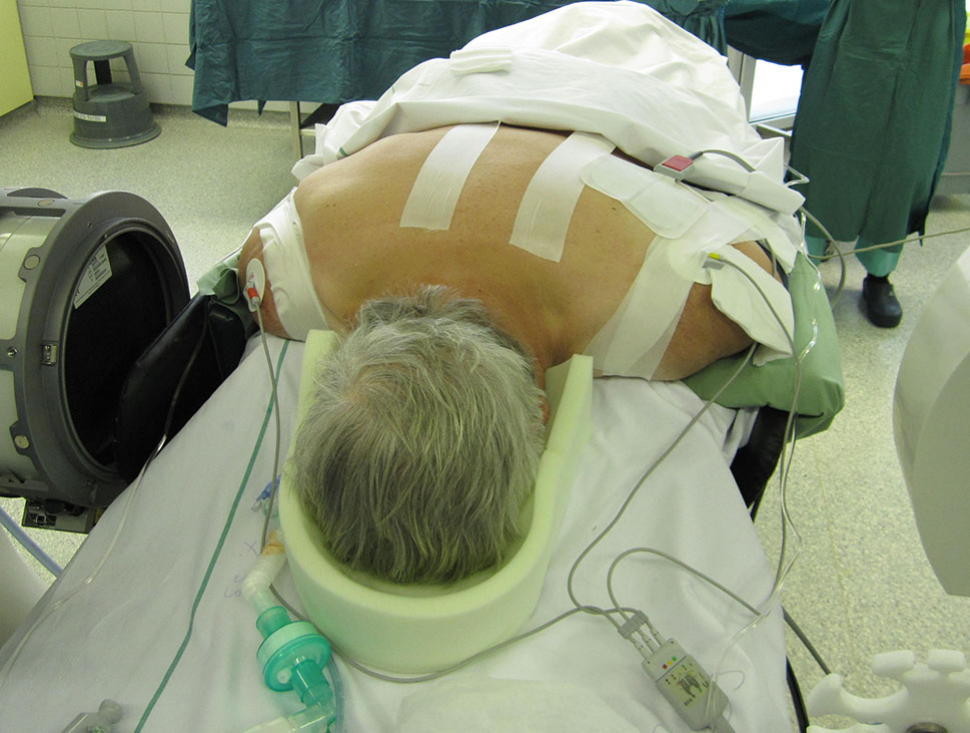
Preoperative picture shows the patient in prone position. Adhesive tapes are used to retract the shoulders caudally for an optimal lateral fluoroscopic view

**Fig. 2:**
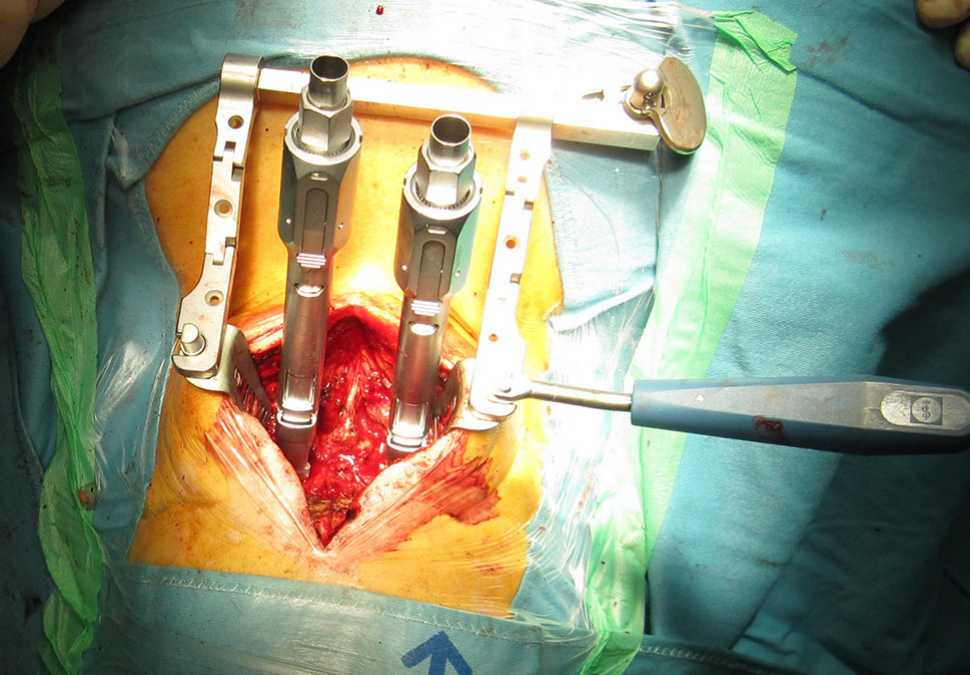
Both pedicle screws are inserted and the reduction extenders are attached

**Fig. 3:**
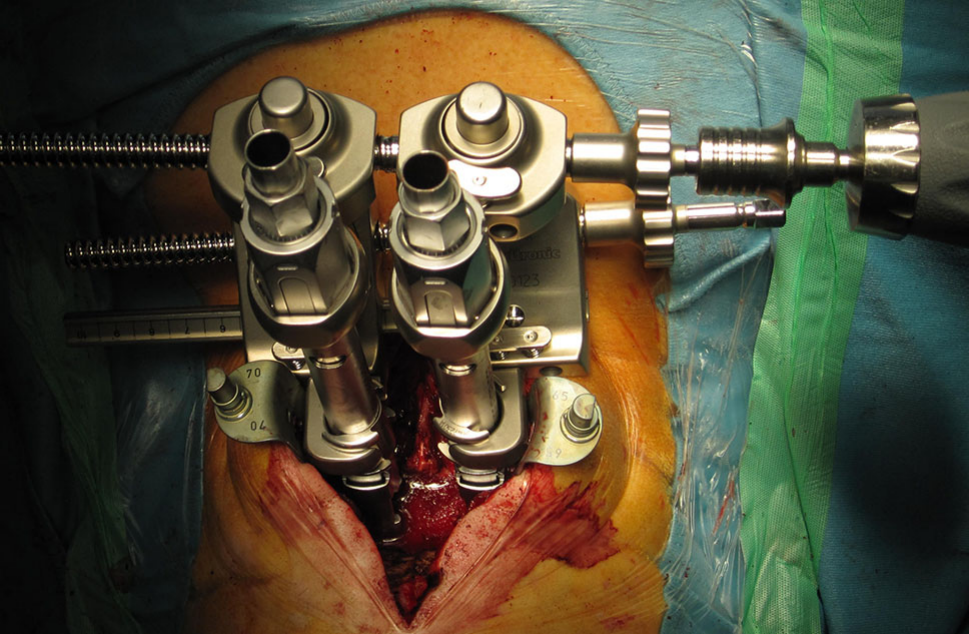
The rod is already inserted. A special reduction device is used to approximate the dislocated lateral masses (posterior reduction). Reduction of the anterior part of the lateral masses happens when reducing monoaxial screws to the rod. Reduction is proofed in the transoral fluoroscopic view

**Fig. 4:**
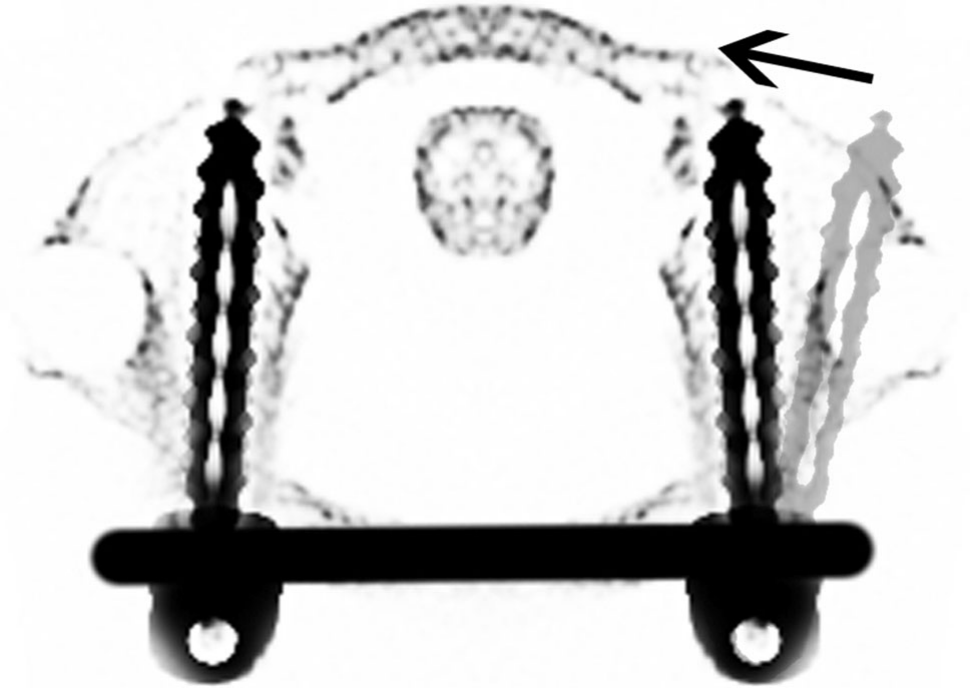
Graphic showing the effect of reducing monoaxial screws to the rod to a perpendicular position

In prone position (Fig. 1), a longitudinal skin incision over the arch of C1 was performed. After dissection of the subcutis and the fascia, the posterior arch of C1 was dissected for direct visualization of the screw entry points. The lateral masses of C1 were probed with machine drilled k-wires using two fluoroscopes with transoral and lateral view. A cannulated drill (2.7 mm) was used to ream the stiff bone near the facette. Length of the pedicle screws was measured and two monoaxial pedicle screws (4.5 mm, CD Horizon Longitude™, Medtronic^®^ Spinal and Biologics Business, 2600 Sofamor Danek Drive, Memphis, TN 38132) were inserted after tapping. By using a straight or slightly kyphotic rod, the fracture was reduced by fixing the rod with the screw nuts (Fig. 2). Additionally, the lateral shift of the lateral mass fragments was reduced with a reduction forceps (Medtronic^®^ Spinal and Biologics Business, 2600 Sofamor Danek Drive, Memphis, TN 38132) (Fig. 3). Due to the monoaxial design of the screws, a perpendicular position of the screw to the rod is achieved when reducing the extender (Fig. 4). Transoral views were used to proof the correct reduction of the dislocated lateral masses of C1. Postoperatively, a CT scan was performed proofing the right screw position. After wound closure, a soft collar was applied for 6 weeks.

### Clinical series

#### Case 1

**Fig. 5:**
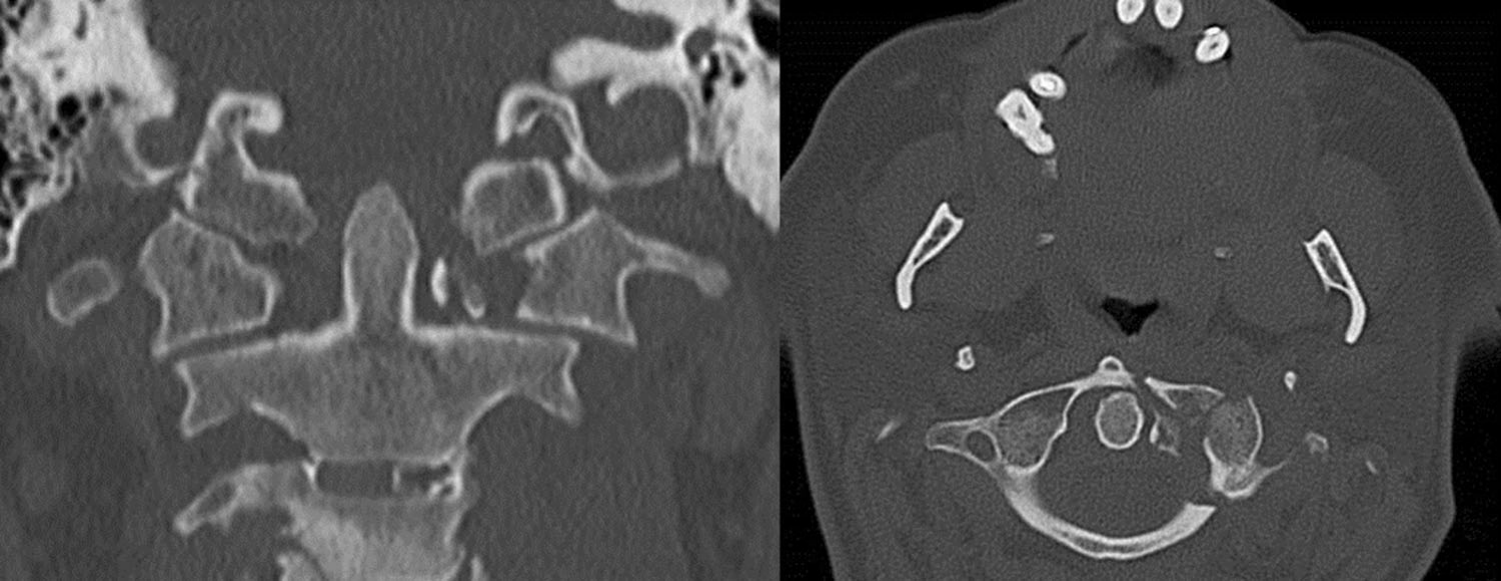
AP (*left*) and transversal (*right*) CT images of the upper C-spine of patient 1. Fracture of the left lateral mass of C1 (Jefferson type IV, Landells and Van Peteghem type III; Fig. 5) with dislocation of 7 mm

**Fig. 6:**
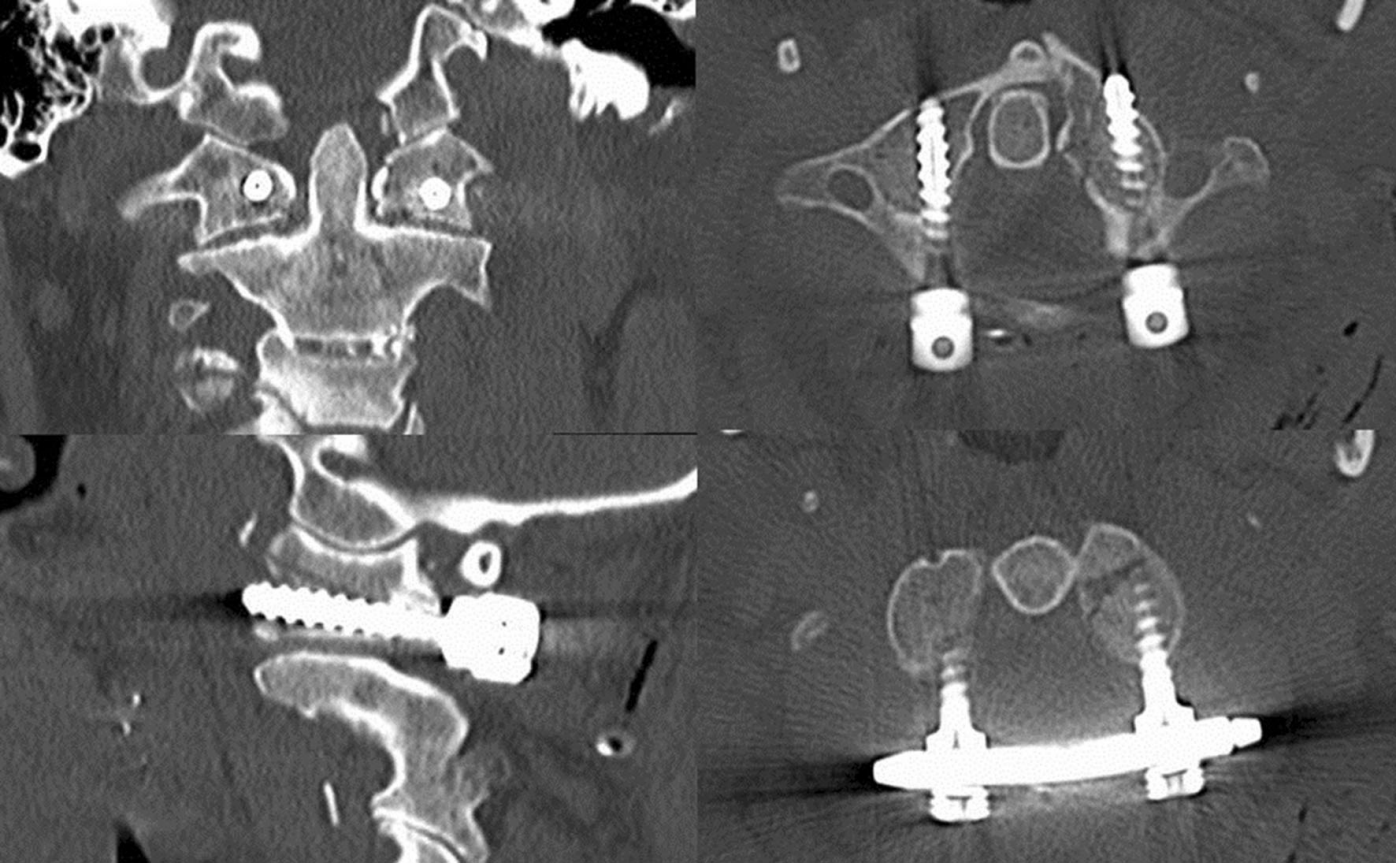
Coronal (*top left*), sagittal (*bottom left*) and transversal (*right*) CT images of patient 1 after ring osteosynthesis. The left lateral mass of C1 is reduced and fixed by monoaxial screws and a rod

A 68-year-old woman felt down form a ladder and complained of headache and pain in the upper neck. She was initially immobilized with a hard collar. CT examination showed a fracture of the base of the skull and a fracture of the left lateral mass of C1 (Jefferson [4] type IV, Landells and Van Peteghem [9] type III; Fig. 5) with dislocation of 7 mm and bony avulsion of the transverse ligament. On day 5, surgery was performed with a mild overcorrection of the left lateral mass (Fig. 6). After 6 weeks, the patient was nearly free of pain. In a CT scan after 11 weeks, the fracture was bony healed and the patient was free of complaints (left rotation of 45°, right rotation 40°, no limitation in inclination). The hardware was removed 14 months later. 10 months after hardware removal, the patient still had no pain. Range of movement was 60° for right rotation, 50° for left rotation and the chin-jugulum distance 1.5 cm in flexion.

#### Case 2

**Fig. 7:**
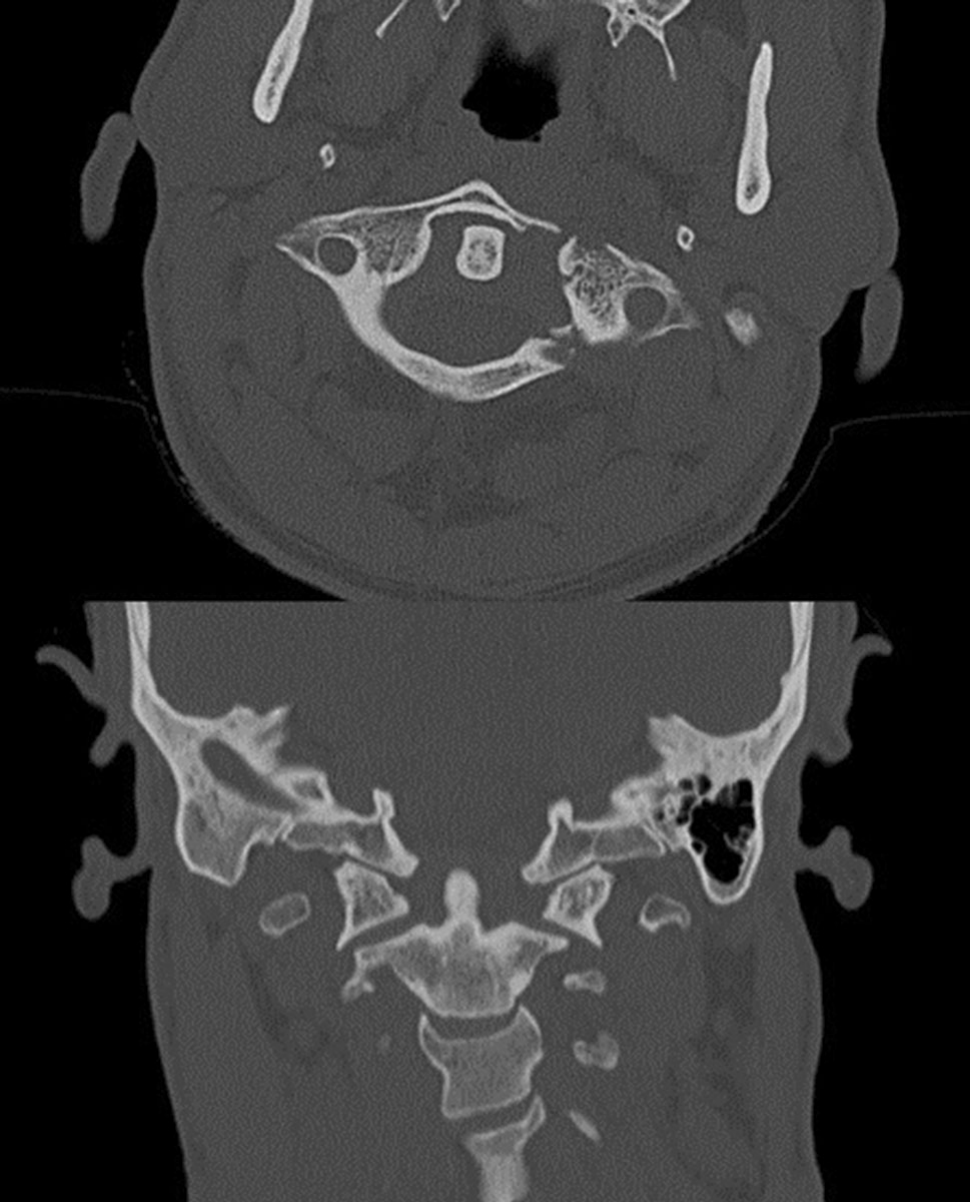
Transversal (*top*) and coronal (*bottom*) CT images of patient 2: Jefferson type III (Landells and Van Peteghem type II) fracture with dislocation of the left lateral mass of approximately 8 mm

**Fig. 8:**
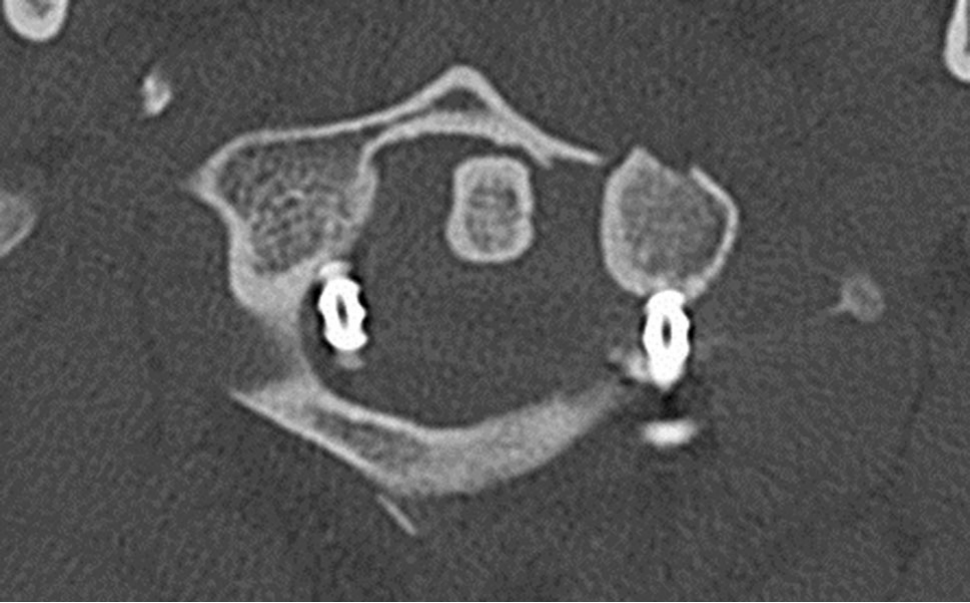
Transversal CT image: medial position of the right lateral mass screw in the spinal canal after surgery (patient 2)

**Fig. 9:**
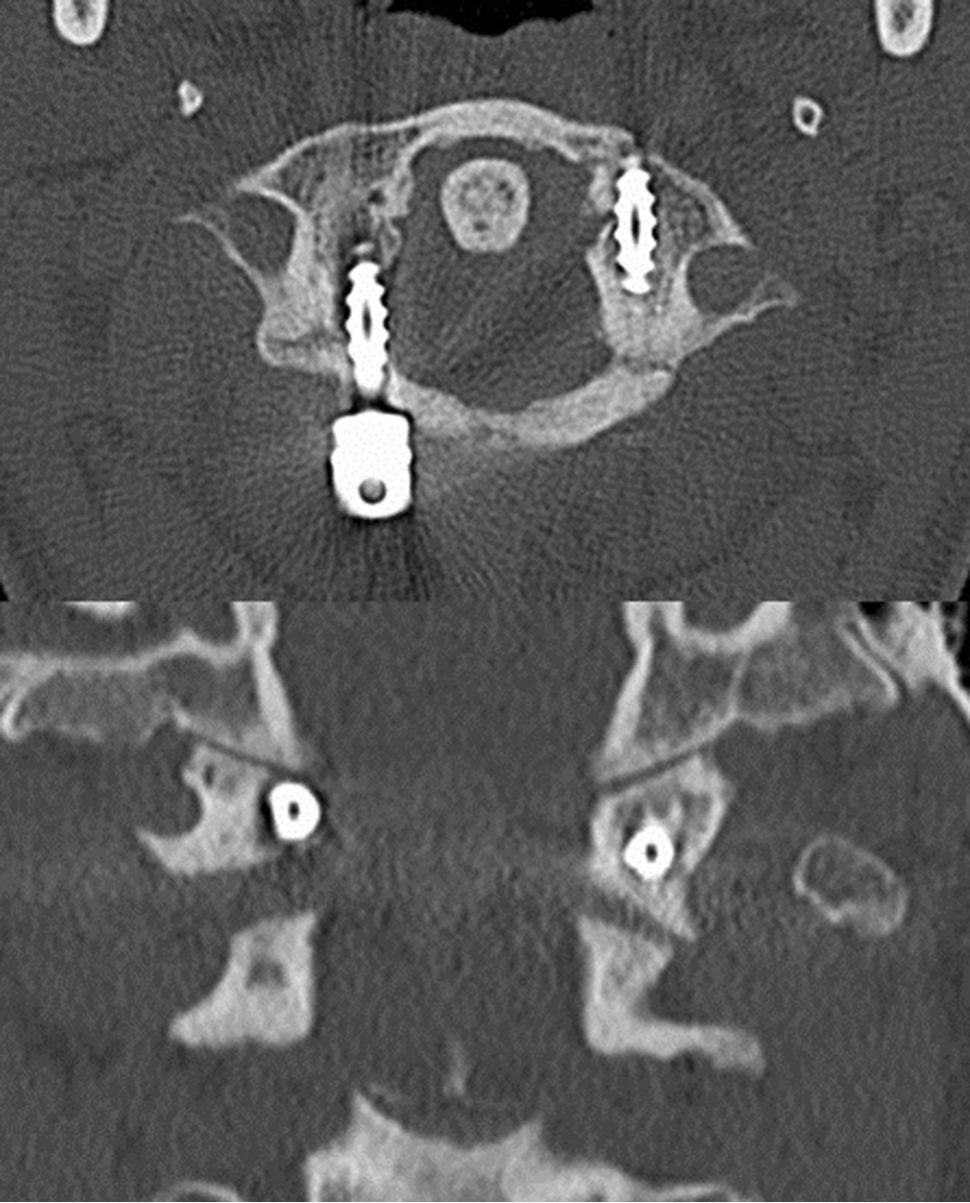
Transversal (*top*) and coronal (*bottom*) CT images of patient 2 after repositioning of the right screw, which is still in a slightly medial but acceptable position. Moderate redislocation of the left lateral mass

A 34-year-old man jumped into shallow water, had initially strong pain in the neck and no neurological deficits. Initial CT examination showed a Jefferson type III (Landells and Van Peteghem type II) fracture with dislocation of the left lateral mass of approximately 8 mm (see Fig. 7). After temporary immobilization with a hard collar surgery was performed on day 4. Postoperatively the patient was re-examined with CT, which showed a medial position of the right lateral mass screw in the spinal canal (see Fig. 8). Although the patient had no neurological deficits, a surgical revision with repositioning of the right screw was performed on the same day. Final CT examination showed a minimal affection of the spinal canal by the screw, but this screw position was tolerated still having no neurological findings (see Fig. 9). In a CT examination after 8 weeks, the left arch was not bony healed yet, the patient had a right and left rotation of 45° in neutral sagittal position. However, he reported minor dysesthesias in the dermatomes of C2 on both the sides. In a CT scan after 4 months, the fracture was bony healed. The hardware removal was performed 5 months later. 21 months later, the dysesthesias occur infrequently requiring no therapy yet, left rotation improved to 90° and right rotation to 70°.

#### Case 3

**Fig. 10:**
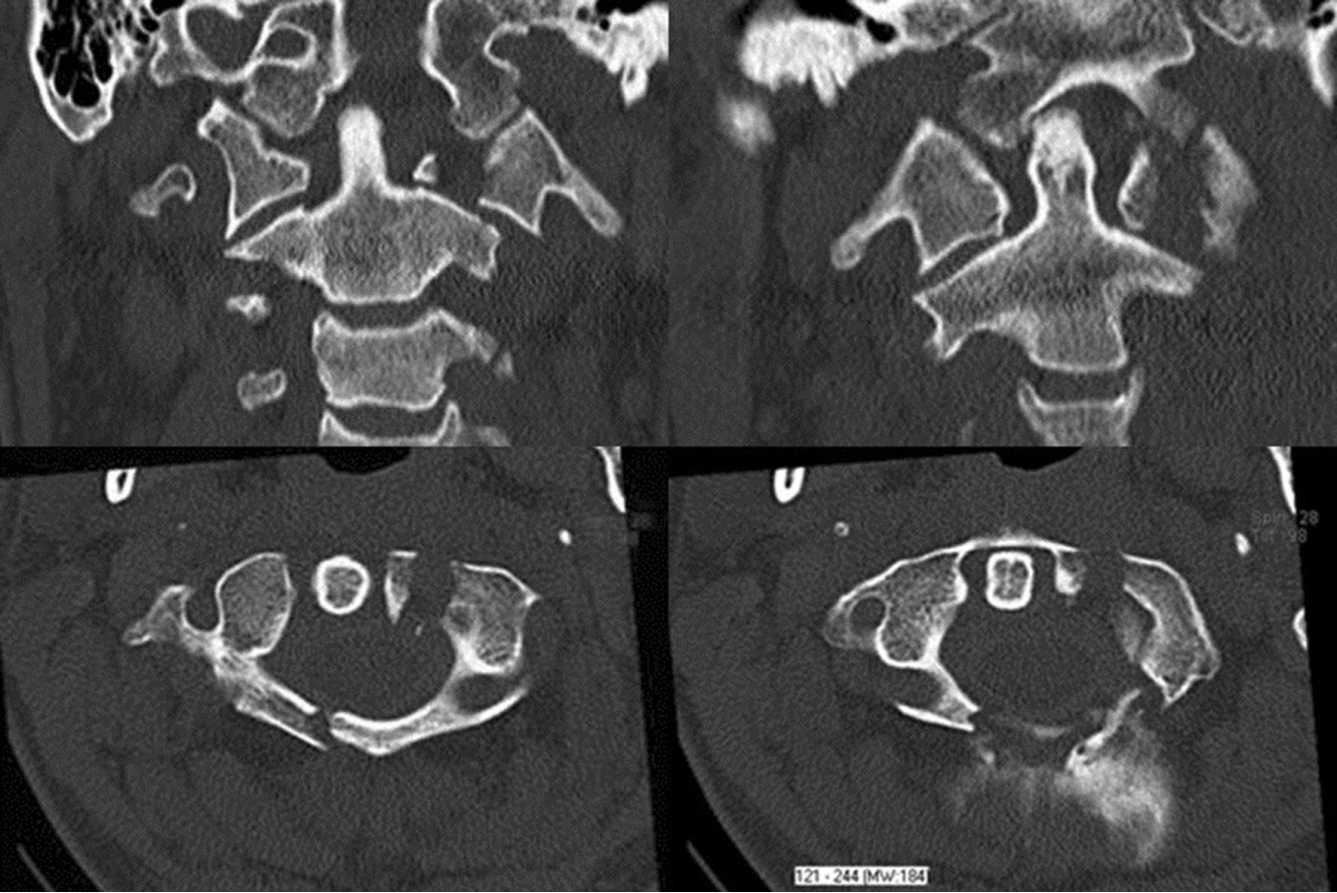
Coronal (*top*) and transversal (*bottom*) CT images of patient 3: Jefferson type IV (Landells and Van Peteghem type III) and posterior arch fracture with dislocation of the left lateral mass

**Fig. 11:**
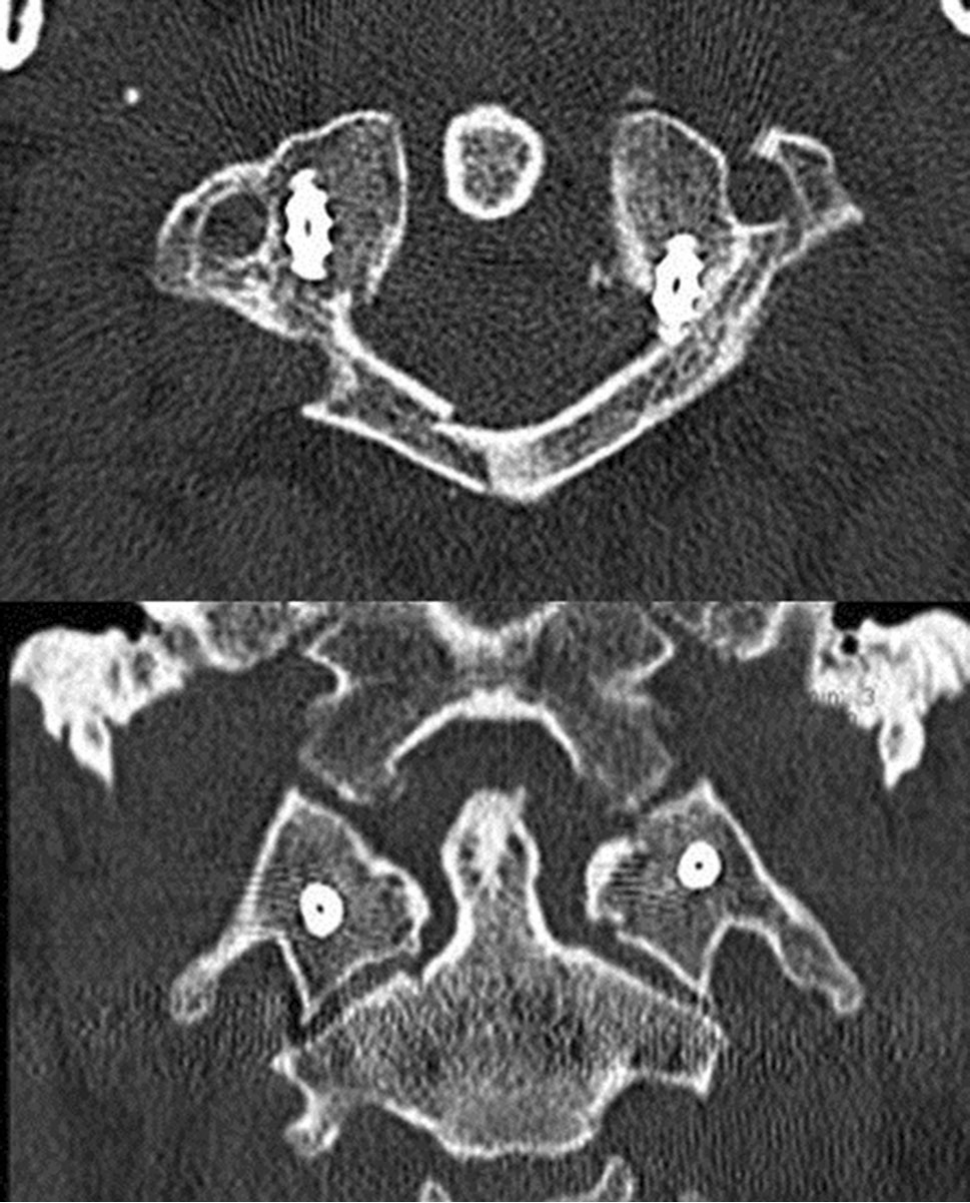
Transversal (*top*) and coronal (*bottom*) CT images of patient 3 after surgery: good reduction of the left lateral mass and closure of the arches

After a skiing accident, a 50-year-old man suffered a slight traumatic brain injury and a Jefferson type IV (Landells and Van Peteghem type III) fracture including the posterior arch with dislocation of the left lateral mass (Fig. 10). Surgery was performed 2 days after the accident. Initial paresthesias of all fingertips disappeared after surgery. Postoperative CT scans showed an ideal position of the screws and a proper reduction (Fig. 11). The patient complained minor bilateral dysesthesias in the dermatomes of C2 after surgery. 3 months later, the fracture was bony healed. There was no need of analgetic drugs anymore. Rotation was limited (20° in both directions) and the chin-jugulum distance was 1 cm. A hardware removal was recommended to the patient but he continued medical treatment in his home country and was therefore lost for follow-up.

**Fig. 12–14:**
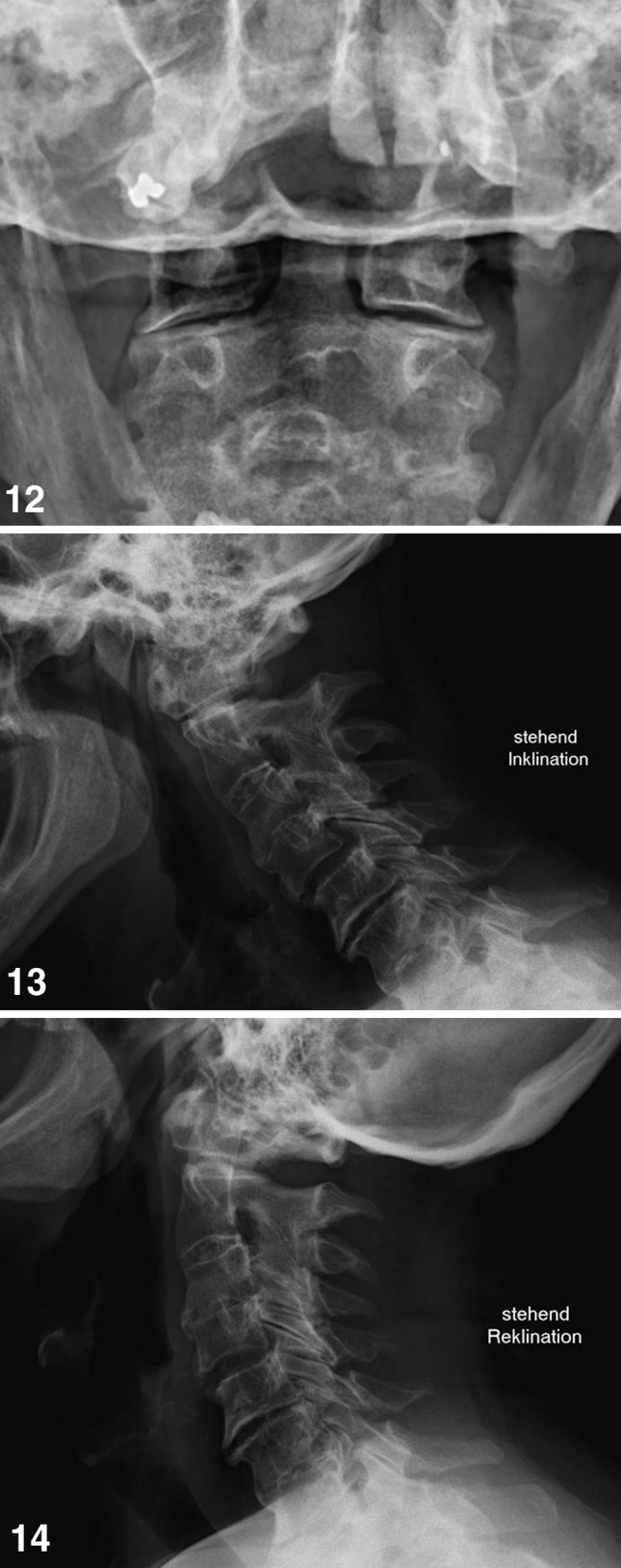
Transoral, lateral flexion and extension views of patient 1, 4 years after surgery

In patient 1, a follow-up examination was performed 5 years after primary surgery. In standard X-ray we found a congruent and stable situation in both joints (Figs. 12, 13, 14) and no complaints in upper cervical spine. The other two patients were lost for long-time follow-up.

## Results

### Clinical assessment/outcome

After hardware removal, two patients had a left rotation in neutral sagittal position of 45°–90°, a right rotation of 45°–70° and distance between chin and jugulum of 0–3 cm.

The third patient was lost for follow-up. All three patients had/had were free of pain after 3 months.

### Radiological assessment

All fractures were bony healed between 11 and 13 weeks. 5 of 6 lateral mass screws were positioned correctly, one screw penetrated into the spinal canal (case 2). In two patients, the fractures were fixed in an anatomically reduced position; in one patient (case 2) we had a loss of reduction of 2 mm after correction of the penetrating screw.

### Further complications

We noticed no problems with wound or bone healing. In case 2, a screw penetrated the spinal canal, and in two of three patients the nerve roots of C2 were irritated after surgery. Hardware removal was performed in two cases without any complications.

## Discussion

Although fixation of Jefferson fractures with pedicle screws is not a new technique, we describe the first cases performed with monoaxial screws [6, 7]. Whereas most isolated C1 ring fractures with mild or no dislocation of the lateral mass can be treated conservatively by a collar or a halo brace for 3 months, there is still discussion about how to treat unstable fractures with dislocation of the lateral mass of more than 7 mm or disruption of the transverse ligament [1, 5]. Some authors still favour a fusion of C1–C2, others are recommending a transoral approach to fix C1 fractures with plates or a rod [8, 10]. The posterior ring osteosynthesis offers enough stability for healing of bony injuries of C1 and should therefore be preferred especially in young patient to preserve the function of the atlantoaxial joint and a physiological range of motion. In patient 1 and 3, we had the situation of a bony avulsion of the transverse ligament—which is—in our opinion—the optimal indication for motion preserving technique. In patient 2, we found no avulsion fragment of the ligament—therefore a rupture has to be assumed. Unfortunately, this patient could not be examined for long term. But what about purely ligamentous injuries? Is a bony ring reduction and fixation sufficient for healing of the transverse ligament? Abelos et al. described 2011 a ring fixation with two polyaxial pedicle screws and a rod stabilizing a Jefferson type III fracture after failed union with halo brace for 3 months [6]. The fracture was healed 7 months after surgery [3]. The use of a halo vest in adults with a C1 ring fracture should be discussed seriously, because stable fractures can be immobilized sufficiently with a collar and unstable fractures require a ring osteosynthesis—when possible—or fusion. Monoaxial screws with the possibility of a reduction of the ventral and the dorsal part of the arches should be preferred when performing a ring osteosynthesis. Opening and reaming of the lateral mass of C1 was one of the difficulties that have to be overcome with the CD Horizon Longitude™ system. This system is designed for thoracic and lumbar spine where the pedicles are probed with a Yamshidi needle. For our purpose, we had to modify the technique by using machine drilled k-wires and cannulated drills (Synthes GmbH, Eimattstraße 3, CH-4436 Oberdorf, Switzerland). The use of an intraoperatively CT scan could avoid screw misplacement harming the spinal canal or the vertebral artery. Greater studies are mandatory to confirm the results of our case series. Monoaxial screws with smaller diameters, smaller sleeves and appropriate reduction tools could facilitate the surgical procedure and reduce the risk of C2 nerve root and other soft tissue irritation.

For future patients, we plan to improve diagnostic and therapeutic procedures. Intraoperative 3D-CT scans are now available at our operating theatre to proof correct position of k-wires before drilling and taping as well as insufficient reduction or overcorrection of the fracture. This procedure could be a powerful alternative technique for bony avulsion fractures of the transverse ligament. But whether a motion preserving technique is able to deal with ligamentous ruptures—ending up in an acceptable tight scar-situation—is highly questionable. Beside clinical outcome measurement, postoperative functional CT scans could perhaps help us to answer this question in future. In these cases, a definitive fusion of C1/2 should be considered as a well-established method.
